# Supplementary material for: SHP-2 and PD-1-SHP-2 signaling regulate myeloid cell differentiation and antitumor responses
Source: Nat Immunol. 2022 Dec 29;24(1):55–68. doi: 10.1038/s41590-022-01385-x (PMC9810534; doi:10.1038/s41590-022-01385-x)
Supplement: Supplementary file 1 — Supplementary Figs. 1–5. [file 41590_2022_1385_MOESM1_ESM.pdf]

nature immunology

Article

https://doi.org/10.1038/s41590-022-01385-x

SHP-2 and PD-1-SHP-2 signaling regulate myeloid cell differentiation and antitumor responses

In the format provided by the authors and unedited

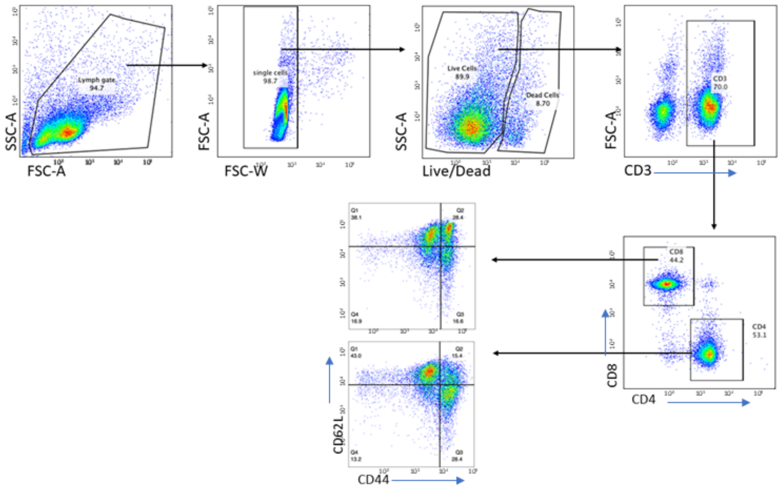

Supplementary Fig. 1. Gating strategy of activated CD4$^+$ and CD8$^+$ T cells in spleen and tumor draining lymph nodes. After excluding doublets and dead cells, CD3$^+$ T cells were divided into CD4$^+$ and CD8$^+$ T cells. Within the CD4$^+$ and CD8$^+$ gates, expression of CD44 and CD62L was assessed. CD4$^+$ and CD8$^+$ in the CD44^{hi}CD62L^{low} gate were defined as T effector (T_{EF}), whereas cells in the CD44$^+$CD62L^{hi} gate were defined as central memory (T_{CM})-like cells.

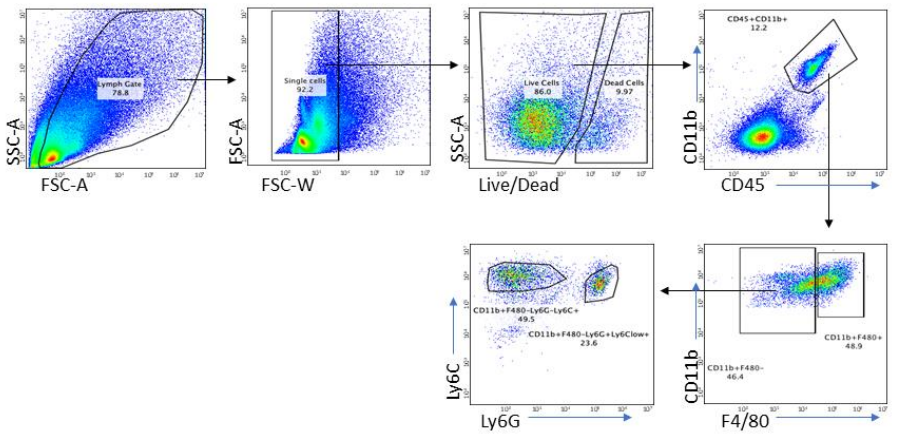

Supplementary Fig. 2: Gating strategy of myeloid subsets in the tumor site. After excluding doublets and dead cells, myeloid cells were identified as CD45^{+}CD11b^{+}. Within the myeloid cell population, macrophages were identified as CD11b^{+}F4/80^{+}, M-MDSCs as CD11b^{+}F4/80^{-}Ly6C^{hi}Ly6G^{-} and PMN-MDSCs as CD11b^{+}F4/80^{-}Ly6C^{lo}Ly6G^{+}. The same gating strategy was used for identification of myeloid subsets in the spleen.

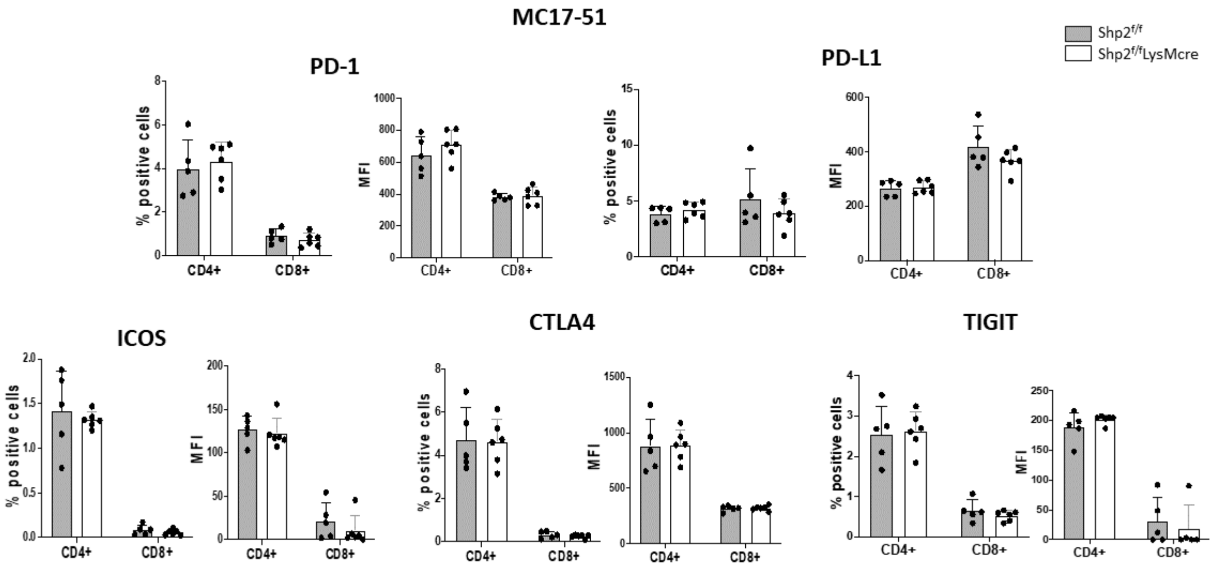

Supplementary Fig. 3. Myeloid-specific SHP-2 deletion does not affect the expression of co-inhibitory receptors in $CD4^{+}$ or $CD8^{+}$ T cells. Expression of the indicated markers was assessed by flow cytometry in T cells isolated from TDLN of *$Shp2^{f/f}$* and *$Shp2^{f/f}LysM^{Cre}$* mice bearing MC17-51 tumors. Representative plots and mean percentages ± SD in *$Shp2^{f/f}$* mice (grey bar) and *$Shp2^{f/f}LysM^{Cre}$* (black bars) are shown. Results are from one representative of four independent experiments with n=5 mice per group.

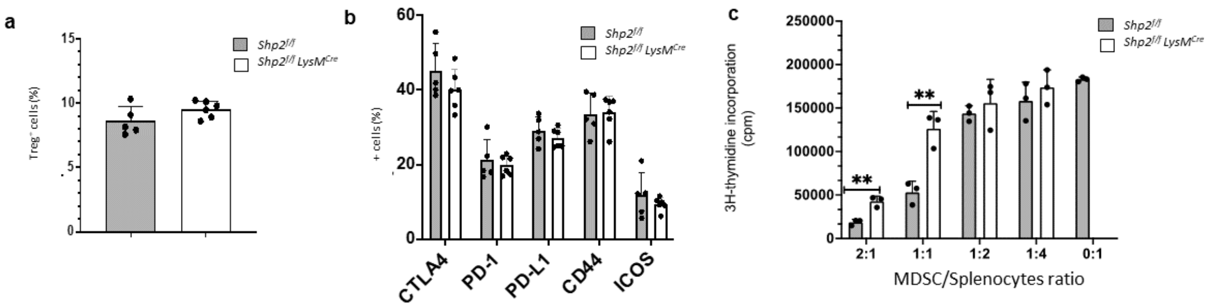

Supplementary Fig. 4. (a, b) Myeloid-specific SHP-2 deletion does not affect the expansion or activation of Treg cells. Tregs were identified by flow cytometry in TDLN of Shp2$^{f/f}$ and Shp2$^{f/f}$LysMCre mice bearing MC17-51 tumors **(a)**. The expansion of the indicated markers on Treg cells was examined **(b)**. Representative results from one of five independent experiments with n=4 to 6 mice per group are shown. **(c) Diminished suppressive activity of M-MDSC isolated from Shp2$^{f/f}$LysMCre tumor-bearing mice**. CD11b^{+}Ly6C^{hi}Ly6G^{-} monocytic (M-MDSC) cells were isolated from *Shp2$^{f/f}$ and Shp2$^{f/f}$LysM^{Cre}* MC17-51 tumor-bearing mice and cultured at various ratios with OTI splenocytes (2x10^{5}cells/well) stimulated with OVA$_{257-264}$ as described in Methods. Mean ± SEM of cpm values are shown. Results are representative of two separate experiments using MDSC from n=3 mice per group. (**p< 0.01).

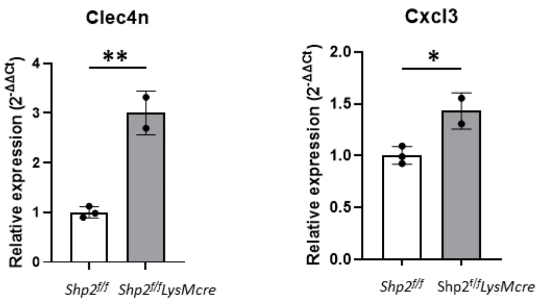

Supplementary Fig. 5. RNAseq validation example. TAMs were isolated from *Shp2*$^{f/f}$ and *Shp2*$^{f/f}$*LysM*^{Cre} tumor-bearing mice on day 15 and expression of mRNA for the indicated genes was examined by qPCR. Results shown are from one of four separate experiments with n=2-3 mice per group. (*$p < 0.05$, **$p < 0.01$).
