# Supplementary material for: SHP-2 and PD-1-SHP-2 signaling regulate myeloid cell differentiation and antitumor responses
Source: Nat Immunol. 2022 Dec 29;24(1):55–68. doi: 10.1038/s41590-022-01385-x (PMC9810534; doi:10.1038/s41590-022-01385-x)
Supplement: Supplementary file 2 — Reporting Summary [file 41590_2022_1385_MOESM2_ESM.pdf]

nature portfolio

Corresponding author(s): Vassiliki A. Boussiotis

Last updated by author(s): Nov 3, 2022

Reporting Summary

Nature Portfolio wishes to improve the reproducibility of the work that we publish. This form provides structure for consistency and transparency in reporting. For further information on Nature Portfolio policies, see our Editorial Policies and the Editorial Policy Checklist.

Statistics

For all statistical analyses, confirm that the following items are present in the figure legend, table legend, main text, or Methods section.

n/a	Confirmed	
☐	☒	The exact sample size (*n*) for each experimental group/condition, given as a discrete number and unit of measurement
☐	☒	A statement on whether measurements were taken from distinct samples or whether the same sample was measured repeatedly
☐	☒	The statistical test(s) used AND whether they are one- or two-sided<br>*Only common tests should be described solely by name; describe more complex techniques in the Methods section.*
☐	☒	A description of all covariates tested
☐	☒	A description of any assumptions or corrections, such as tests of normality and adjustment for multiple comparisons
☐	☒	A full description of the statistical parameters including central tendency (e.g. means) or other basic estimates (e.g. regression coefficient) AND variation (e.g. standard deviation) or associated estimates of uncertainty (e.g. confidence intervals)
☐	☒	For null hypothesis testing, the test statistic (e.g. *F*, *t*, *r*) with confidence intervals, effect sizes, degrees of freedom and *P* value noted<br>*Give P values as exact values whenever suitable.*
☒	☐	For Bayesian analysis, information on the choice of priors and Markov chain Monte Carlo settings
☒	☐	For hierarchical and complex designs, identification of the appropriate level for tests and full reporting of outcomes
☒	☐	Estimates of effect sizes (e.g. Cohen's *d*, Pearson's *r*), indicating how they were calculated

Our web collection on statistics for biologists contains articles on many of the points above.

Software and code

Policy information about availability of computer code

Data collection	Flow cytometry data were analyzed with Flowjo 10.4 Software. For Metabolite analysis, polar metabolites were quantitatively profiled by a positive/negative ion–switching, targeted liquid chromatography tandem mass spectrometry (LC-MS/MS) based metabolomics platform using a 5500 QTRAP hybrid triple quadruple mass spectrometer (AB/SCIEX) via selected reaction monitoring (SRM). Once the SRM data for ~285 metabolites were acquired, peaks were integrated using a software platform for peak area integration MultiQuant 2.1 (AB/SCIEX). Data analysis was performed using online MetaboAnalyst 3.0 software. Raw sequencing reads were quality-checked using FastQC (v0.11.5) and data were pre-processed with Cutadapt (v2.5) for adapter removal following best practices. Gene expression quantification was performed by aligning against the GRCm38 genome using STAR (v2.7.3aand quantifying reads against Ensembl v98 annotated gene loci with featureCounts (Subread 1.6.2). Differential gene expression analysis was performed using DESeq2 (v1.24.0), while ClusterProfiler (v3.12.0) was utilized for downstream functional investigations. Plots were generated in R using ggplot2 (v3.3.3), EnhancedVolcano (v1.8.0), and ComplexHeatmap (v2.6.2). Storey's q value was utilized to control family-wise error rate. All other statistical analyses were performed using GraphPad Prism (GraphPad Software v.9.4.0)
Data analysis	*Provide a description of all commercial, open source and custom code used to analyse the data in this study, specifying the version used OR state that no software was used.*

For manuscripts utilizing custom algorithms or software that are central to the research but not yet described in published literature, software must be made available to editors and reviewers. We strongly encourage code deposition in a community repository (e.g. GitHub). See the Nature Portfolio guidelines for submitting code & software for further information.

Data

Policy information about availability of data

All manuscripts must include a data availability statement. This statement should provide the following information, where applicable:

- Accession codes, unique identifiers, or web links for publicly available datasets
- A description of any restrictions on data availability
- For clinical datasets or third party data, please ensure that the statement adheres to our policy

All data generated or analyzed during this study are included in this published article (and its supplementary information files). Sequencing data have been deposited at the Gene Expression Omnibus database under the accession numbers GSE187394 and GSE206207 and are publicly available.

Human research participants

Policy information about studies involving human research participants and Sex and Gender in Research.

Reporting on sex and gender	N/A
Population characteristics	N/A
Recruitment	N/A
Ethics oversight	N/A

Note that full information on the approval of the study protocol must also be provided in the manuscript.

Field-specific reporting

Please select the one below that is the best fit for your research. If you are not sure, read the appropriate sections before making your selection.

☒ Life sciences ☐ Behavioural & social sciences ☐ Ecological, evolutionary & environmental sciences

For a reference copy of the document with all sections, see nature.com/documents/nr-reporting-summary-flat.pdf

Life sciences study design

All studies must disclose on these points even when the disclosure is negative.

Sample size	No statistical methods were used to predetermine sample sizes, and our sample sizes were similar to those reported in previous publications. Strauss, L. et al. Targeted deletion of PD-1 in myeloid cells induces antitumor immunity. Sci Immunol 5, doi:10.1126/sciimmunol.aay1863 (2020). Strauss, L. et al. RORC1 Regulates Tumor-Promoting "Emergency" Granulo-Monocytopoiesis. Cancer cell 28, 253-269, doi:10.1016/j.ccell.2015.07.006 (2015). Molgora, M. et al. TREM2 Modulation Remodels the Tumor Myeloid Landscape Enhancing Anti-PD-1 Immunotherapy. Cell 182, 886-900 e817, doi:10.1016/j.cell.2020.07.013 (2020).
Data exclusions	No datapoints were excluded from the analyses.
Replication	All experiments were reproducible and were repeated at least three times
Randomization	Mice were assigned randomly to the various experimental groups described. Equal numbers of male and female mice were used in all experiments.
Blinding	Blinding was not used because the studies involved several genetically engineered mouse strains which are hard to breed in high numbers. For this reason, every available mouse should be used judiciously.

Reporting for specific materials, systems and methods

We require information from authors about some types of materials, experimental systems and methods used in many studies. Here, indicate whether each material, system or method listed is relevant to your study. If you are not sure if a list item applies to your research, read the appropriate section before selecting a response.

Materials & experimental systems

n/a	Involved in the study
☐	☐ Antibodies
☐	☒ Eukaryotic cell lines
☒	☐ Palaeontology and archaeology
☐	☒ Animals and other organisms
☒	☐ Clinical data
☒	☐ Dual use research of concern

Methods

n/a	Involved in the study
☒	☐ ChIP-seq
☐	☒ Flow cytometry
☒	☐ MRI-based neuroimaging

Antibodies

Antibodies used	All antibodies used are described in the methods section and in a supplementary table (Supplementary Table 7). All antibodies were used according to the recommendations of the manufacturer unless indicated otherwise.
Validation	*Describe the validation of each primary antibody for the species and application, noting any validation statements on the manufacturer's website, relevant citations, antibody profiles in online databases, or data provided in the manuscript.*

Eukaryotic cell lines

Policy information about cell lines and Sex and Gender in Research

Cell line source(s)	B16-F10 and MC17-51 cell lines were used and were obtained from ATCC.
Authentication	Authentication is provided by the vendor
Mycoplasma contamination	All cell lines were tested negative for mycoplasma contamination. In our laboratory, we perform a regular screening for mycoplasma on a monthly basis.
Commonly misidentified lines (See ICLAC register)	N/A

Animals and other research organisms

Policy information about studies involving animals; ARRIVE guidelines recommended for reporting animal research, and Sex and Gender in Research

Laboratory animals	Mouse
Wild animals	N/A
Reporting on sex	Equal number of male and female mice were uses assigned in all experimental groups. This approach was employed because, the study does not involve a disease with distinct prevalence between sex groups.
Field-collected samples	N/A
Ethics oversight	All mice procedures were approved by the Institutional Animal Care and Use Committee (IACUC) at Beth Israel Deaconess Medical Center (Boston MA), and were in accordance with National Institutes of Health Guidelines for the Care and Use of Animals.

Note that full information on the approval of the study protocol must also be provided in the manuscript.

Flow Cytometry

Plots

Confirm that:

☒ The axis labels state the marker and fluorochrome used (e.g. CD4-FITC).

☒ The axis scales are clearly visible. Include numbers along axes only for bottom left plot of group (a 'group' is an analysis of identical markers).

☒ All plots are contour plots with outliers or pseudocolor plots.

☒ A numerical value for number of cells or percentage (with statistics) is provided.

Methodology

Sample preparation	Samples were prepares from live animals.

Instrument	Flow cytometry samples were acquired sing Becton Dickinson LSR Fortsessa or Beckman-Coulter Cytoflex For Metabolite analysis, polar metabolites were quantitatively profiled by a positive/negative ion–switching, targeted liquid chromatography tandem mass spectrometry (LC-MS/MS) based metabolomics platform using a 5500 QTRAP hybrid triple quadruple mass spectrometer (AB/SCIEX) via selected reaction monitoring (SRM). Once the SRM data for ~285 metabolites were acquired, peaks were integrated using a software platform for peak area integration MultiQuant 2.1 (AB/SCIEX). Raw sequencing reads were quality-checked using FastQC (v0.11.5) and data were pre-processed with Cutadapt (v2.5) for adapter removal following best practices. Gene expression quantification was performed by aligning against the GRCm38 genome using STAR (v2.7.3aand quantifying reads against Ensembl v98 annotated gene loci with featureCounts (Subread 1.6.2). Differential gene expression analysis was performed using DESeq2 (v1.24.0), while ClusterProfiler (v3.12.0) was utilized for downstream functional investigations. Plots were generated in R using ggplot2 (v3.3.3), EnhancedVolcano (v1.8.0), and ComplexHeatmap (v2.6.2). Storey's q value was utilized to control family-wise error rate. Gene sets used for Gene Set Enrichment. Signals on wester blots were visualized, acquired and quantified with Li-COR Odyssey CLx imaging system. For assessment of cell proliferation 3H-thymidine incorporation was measured using a MicroBeta plate counter was used (TriLux Perkin Elmer).
Software	Cells were acquired using Becton Dickinson LSR Fortsessa or Beckman-Coulter Cytoflex Flow cytometer and analyzed with Flowjo Software.
Cell population abundance	No cell sorting was used in any of the experiments
Gating strategy	All detailed relevant information regarding gatingstrategyis provided in Supplementary figures.

☒ Tick this box to confirm that a figure exemplifying the gating strategy is provided in the Supplementary Information.
