# Supplementary material for: SHP-2 and PD-1-SHP-2 signaling regulate myeloid cell differentiation and antitumor responses
Source: Nat Immunol. 2022 Dec 29;24(1):55–68. doi: 10.1038/s41590-022-01385-x (PMC9810534; doi:10.1038/s41590-022-01385-x)
Supplement: Source Data Fig. 7 — Unprocessed western blots. [file 41590_2022_1385_MOESM6_ESM.pdf]

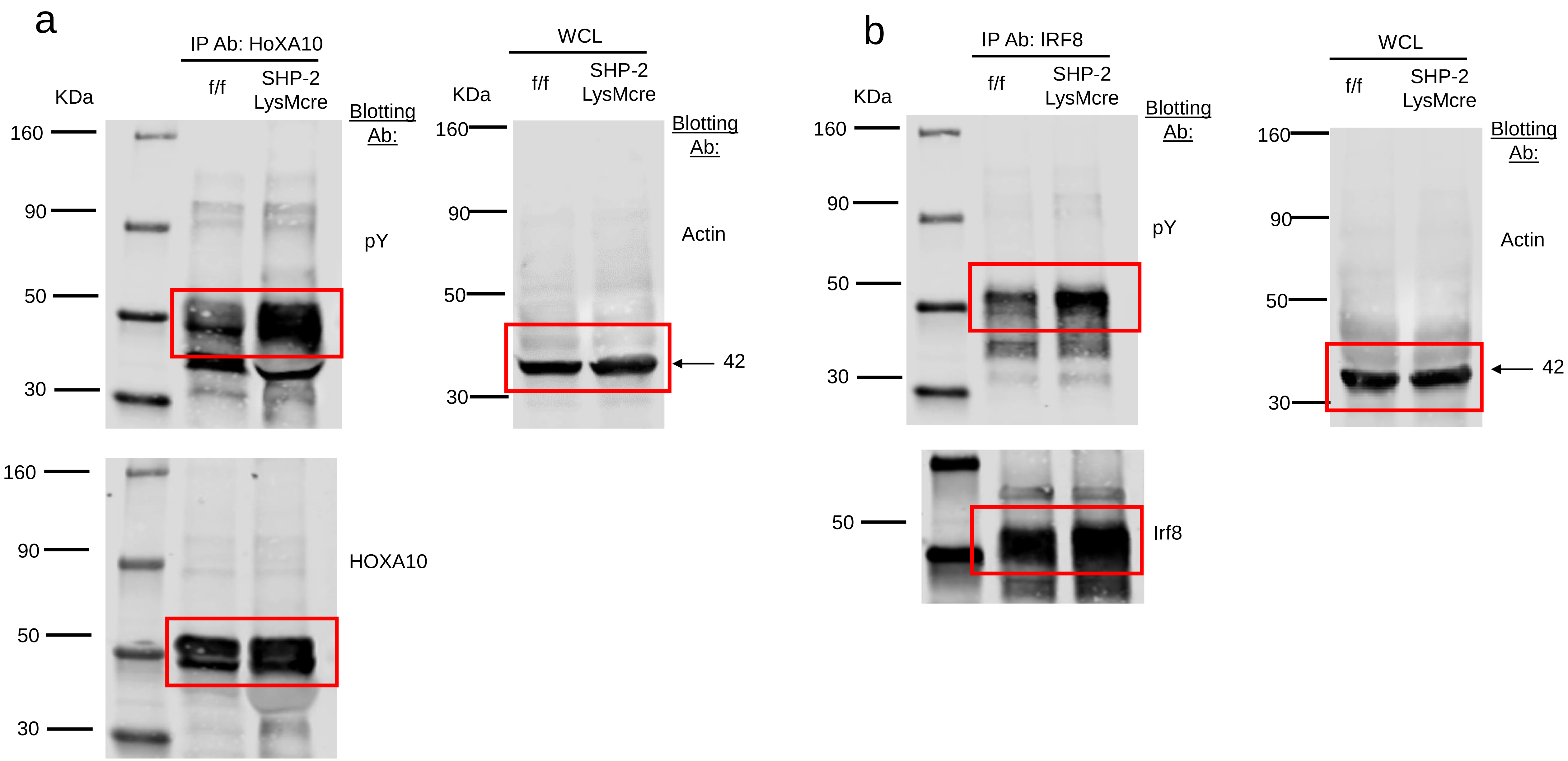

Unprocessed blots Figure 7a

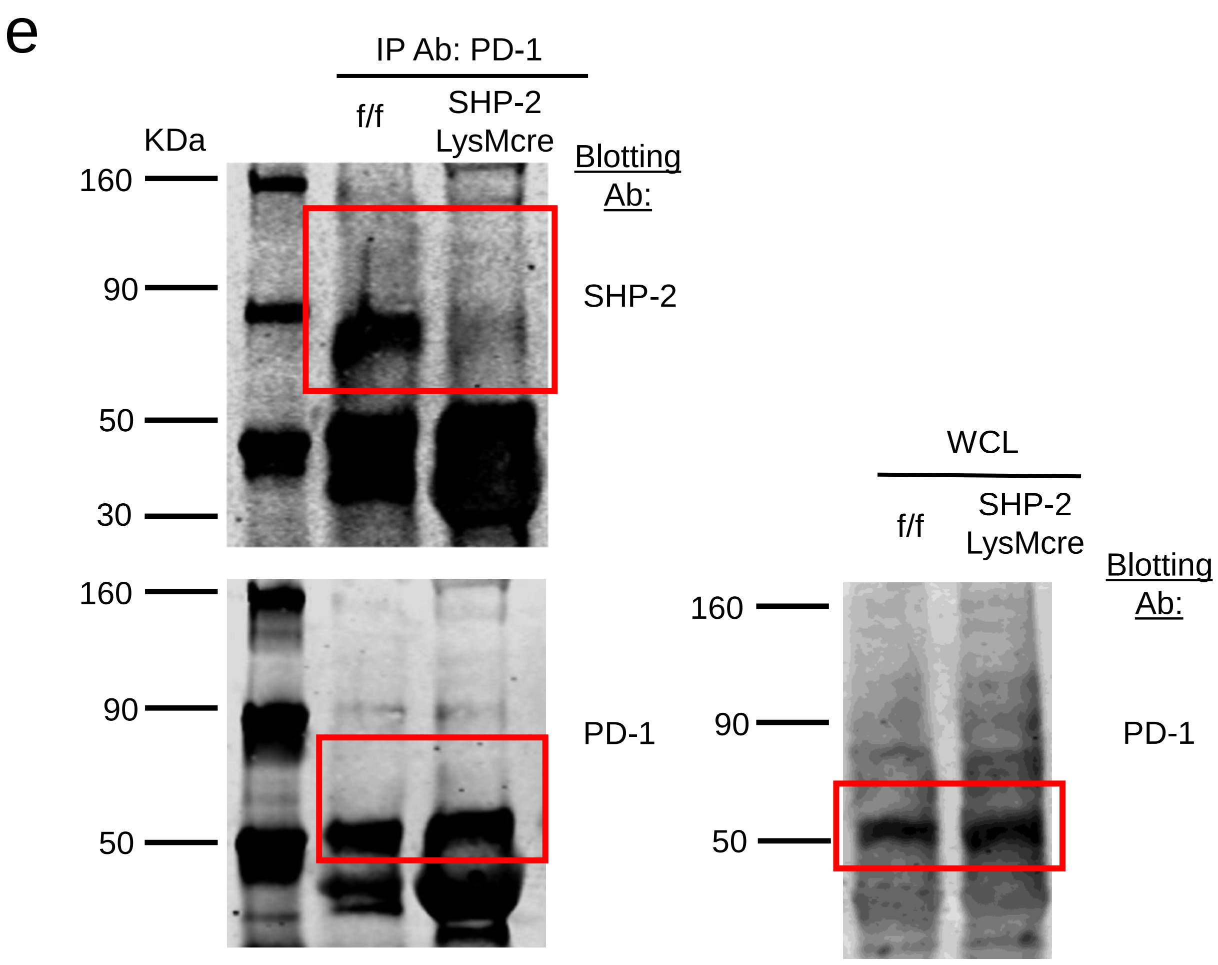

Unprocessed blots 7a

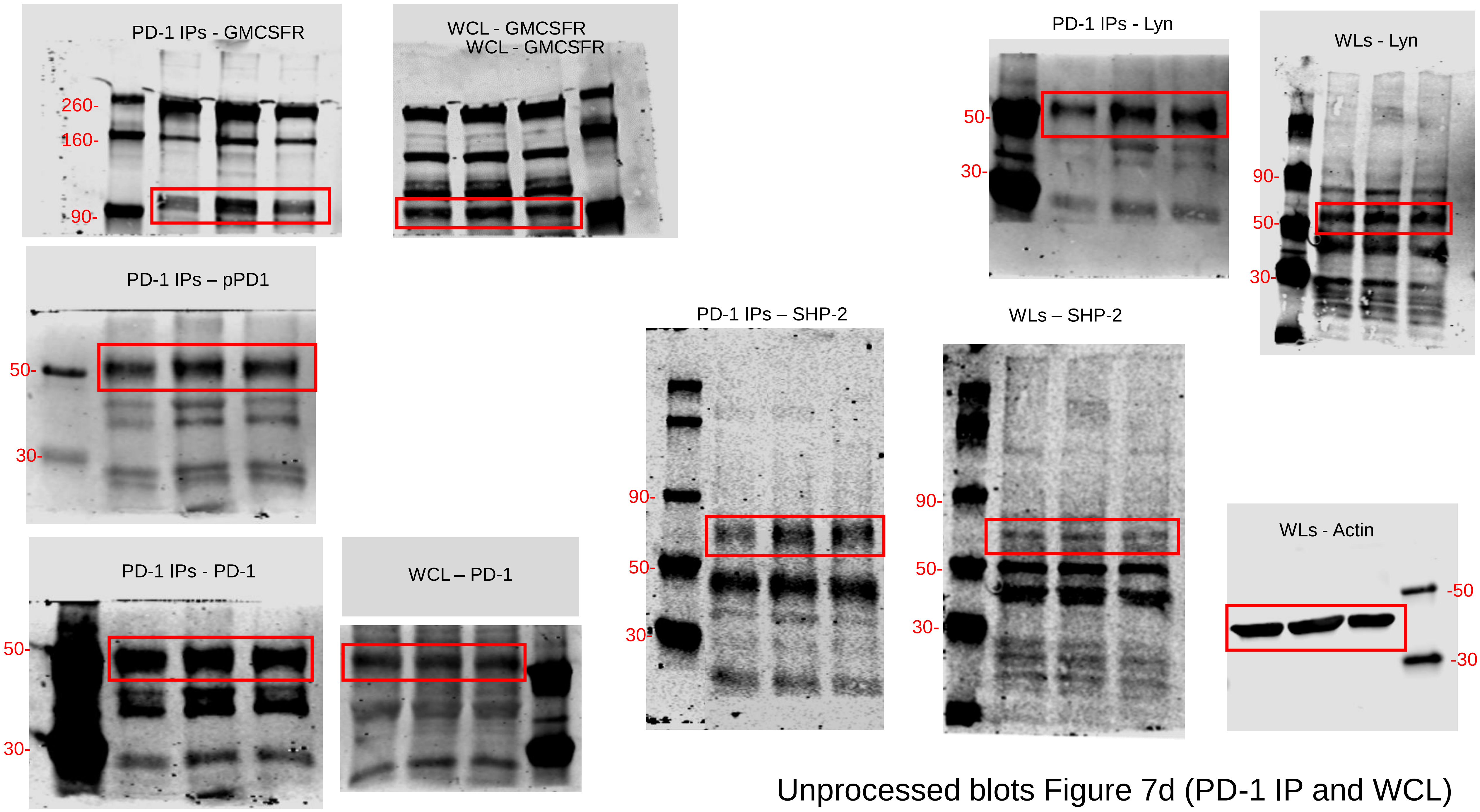

Unprocessed blots Figure 7d (PD-1 IP and WCL)

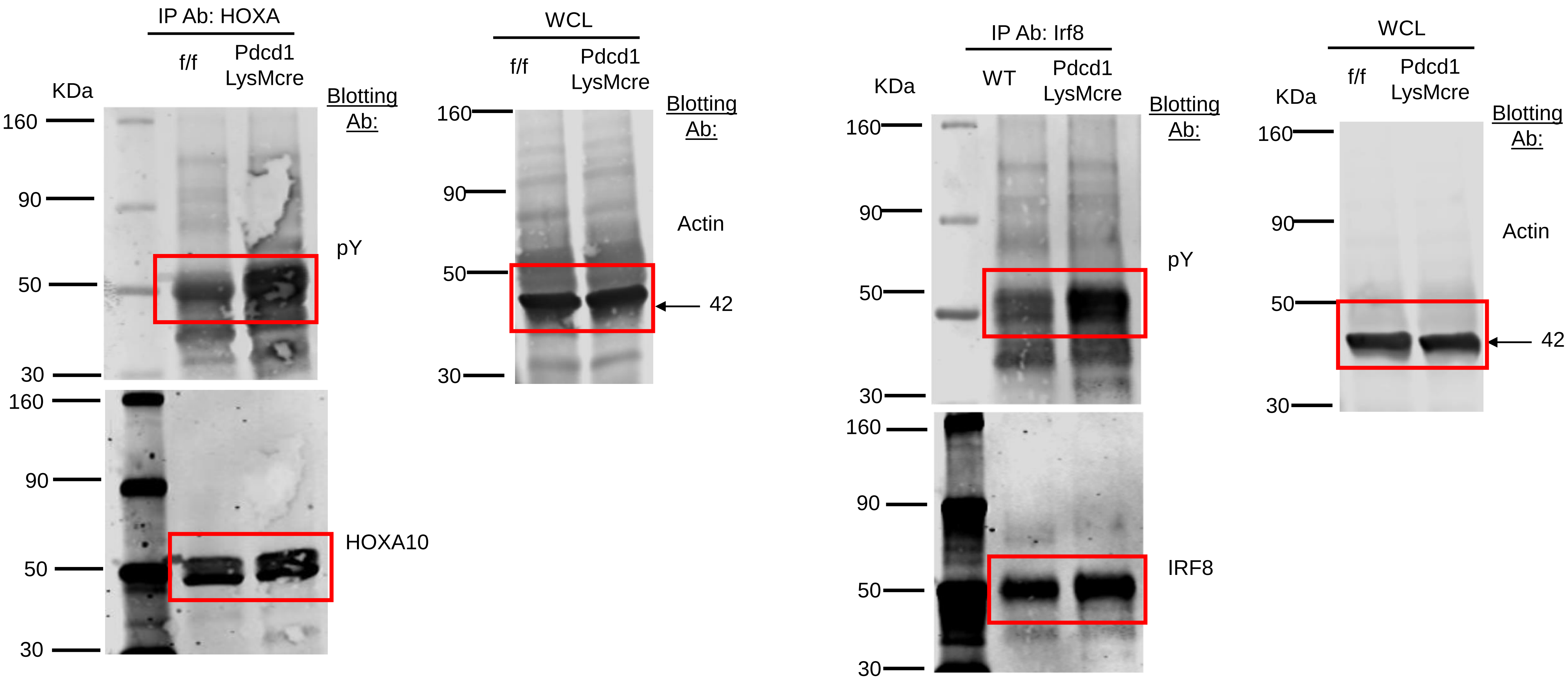

Unprocessed blots Figure 7f
