# Supplementary figures and images for: SHP-2 and PD-1-SHP-2 signaling regulate myeloid cell differentiation and antitumor responses
Source: Nat Immunol. 2022 Dec 29;24(1):55–68. doi: 10.1038/s41590-022-01385-x (PMC9810534; doi:10.1038/s41590-022-01385-x)

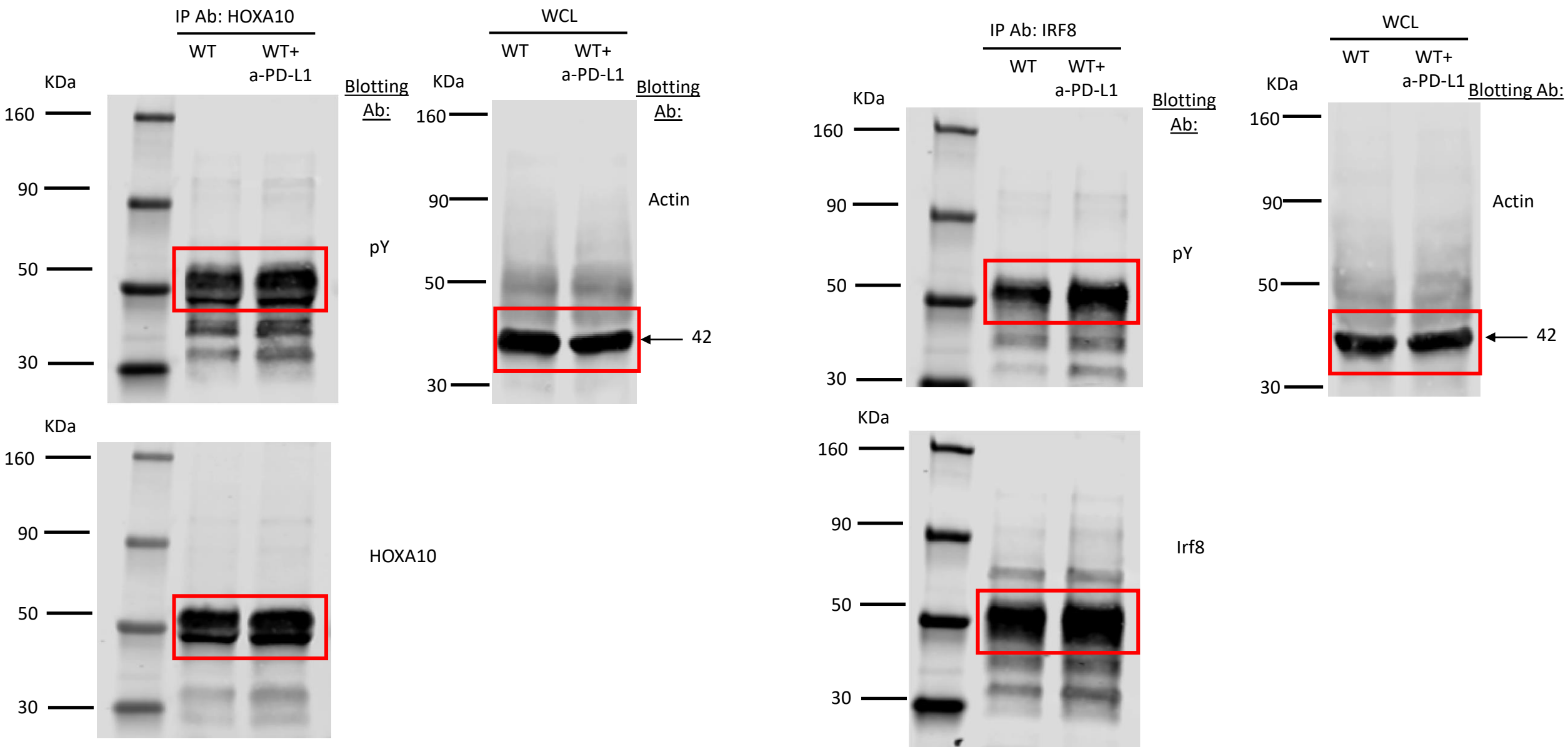

Unprocessed blots Extended Data Fig. 7

Supplement: Source Data Extended Data Fig. 7 — Unprocessed western blots. [file 41590_2022_1385_MOESM7_ESM.pdf]
